# A High-Efficiency Dual-Polarized Transmitarray Antenna with Flat Gain Performance

**DOI:** 10.3390/mi17060637

**Published:** 2026-05-22

**Authors:** Xin-Hui Jiao, Li Zhang, Yu Zhang

**Affiliations:** 1 Institute of Information Sensing, Xidian University, Xi’an 710071, China; 2 School of Electronic Engineering, Xidian University, Xi’an 710071, China; lizhang@mail.xidian.edu.cn (L.Z.); yuzhang@mail.xidian.edu.cn (Y.Z.)

**Keywords:** transmitarray antenna, subwavelength element, high efficiency, flat gain, dual-polarization

## Abstract

This article presents a high-efficiency dual-polarized transmitarray antenna which achieves flat gain characteristics. First, a wideband triple-layer subwavelength element is designed, achieving high transmission amplitude and a full 360° transmission phase range at 10 GHz. To enhance the passband, an isolated element and a cross-shaped middle layer are incorporated. Additionally, four vias connect the top and bottom layers to induce the current resonance, extending the phase range to 360° and improving the radiation efficiency. Based on the element, a transmitarray prototype with 185 elements is fabricated and measured, showing a gain of 27.1 dBi at the center frequency 10.2 GHz, with a radiation efficiency of 58.9%, and a 0.5 dB gain bandwidth of 12.7%. Within the 1 dB gain bandwidth, a minimum radiation efficiency of 37% is achieved at 11.4 GHz.

## 1. Introduction

Transmitarray antennas have attracted increasing attention in multiple application areas such as satellite communications and radar systems owing to their advantages such as high gain, low cost, compact structure, and ease of fabrication [1,2,3]. However, their practical applications are still limited by their narrow operating bandwidth and insufficient radiation efficiency [4]. Accordingly, the design of dual-polarized transmitarrays with wide bandwidth and high efficiency remains an important topic in antenna engineering.

The flat-gain performance is characterized by the 0.5 dB gain bandwidth, within which the gain fluctuation remains within 0.5 dB of the peak value. Compared with conventional wideband designs, the flat-gain transmitarrays are much more difficult to realize, since they require not only sufficient phase coverage, but also stable transmission amplitude and phase responses over frequency. In recent years, several flat gain transmitarrays have been reported [5,6,7,8]. For example, a 1-bit transmitarray with a 37% 1 dB gain bandwidth was presented in [5], while the high-efficiency flat-gain designs with 16.9% 1 dB and 41% 0.5 dB gain bandwidths were reported in [6,7], respectively. Nevertheless, these studies are mainly limited to single linear polarization, and the simultaneous realization of dual polarization, high efficiency, and flat-gain performance remains insufficiently explored.

To improve the bandwidth and efficiency of transmitarrays, two major categories of element designs, namely the current-resonant elements using metallic vias [9,10,11,12] and the multi-layer frequency-selective surface (M-FSS) elements [13,14,15,16], have been widely investigated. The current-resonant elements can usually provide a full 360° transmission phase range, but their bandwidths are often limited due to strong frequency dispersion [12]. By contrast, the M-FSS elements generally provide broader passbands, but their available phase ranges within the low-loss transmission regions are often insufficient [17]. In [18], a wideband high-efficiency transmitarray using two types of compact elements was proposed, and a 1 dB gain bandwidth of 20.4% with 52.5% radiation efficiency is achieved. Meanwhile, most reported dual-polarized transmitarrays primarily focus on bandwidth enhancement, polarization diversity, or efficiency improvement, rather than explicitly targeting the flat-gain performance over frequency. These facts indicate that neither single-resonance mechanism alone can simultaneously satisfy the requirements of wideband phase control and stable transmission characteristics. Therefore, for the flat-gain transmitarrays, it is essential to develop the elements with diverse and complementary resonant properties so that wideband transmission stability and sufficient phase controllability can be achieved simultaneously.

In our previous work [19], the isolated metal elements were introduced to enhance the transmitarray bandwidth by suppressing the inter-element coupling and introducing the capacitive gaps. That design demonstrated an effective improvement in bandwidth and radiation efficiency for a dual-polarized transmitarray. However, the study mainly focused on wideband and high-efficiency performance without addressing the gain variation across frequency. In practice, the flat-gain performance imposes more stringent requirements on both element design and array arrangement, and thus cannot be achieved by directly extending the previous design.

In this work, a high-efficiency dual-polarized transmitarray antenna with flat-gain performance is proposed. First, a triple-layer subwavelength element with enhanced multi-resonant behavior is developed to improve the transmission stability and phase controllability. Then, a phase partition-based array arrangement strategy is introduced, in which the elements are distributed according to both their transmission characteristics and the aperture illumination profile. By combining the multi-resonant element design with the array-level phase partition strategy, the proposed transmitarray simultaneously achieves dual polarization, high efficiency, and flat-gain performance. Compared with our previous work, the present design differs not only in using a triple-layer subwavelength element instead of a two-layer isolated element, but also in its introduction of a phase-partition strategy to realize the quantified flat-gain performance at the array level.

## 2. Design of the Transmitarray Element

The operating mechanism of the proposed element can be understood from the viewpoints of bandwidth formation and phase-range extension. According to Ref. [16], adopting a middle layer with a topology different from that of the outer layers can introduce additional resonant freedom and thereby broaden the transmission passband. In addition, as demonstrated in [19], electrically decoupling adjacent elements can suppress the inter-element current coupling, while the introduced capacitive gaps provide additional degrees of freedom for the transmission-response engineering. This feature is beneficial for bandwidth enhancement compared with conventional connected structures.

**Figure 1:**
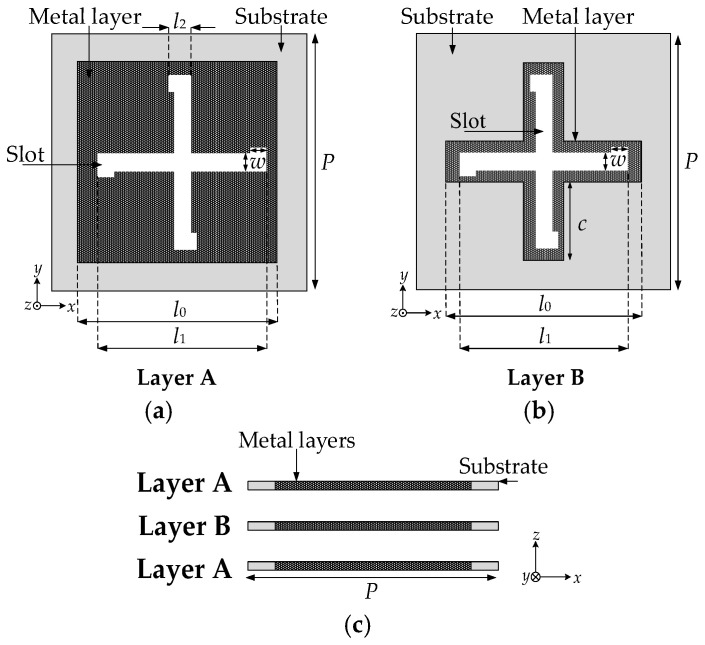
Configuration of the triple-layer element. (**a**) Top view of Layer A; (**b**) top view of Layer B; and (**c**) side view.

Motivated by these observations, the slot-based triple-layer structure shown in Figure 1 is first constructed, in which the transmission response is mainly governed by the slot-related resonances. Two structural features are introduced to establish the broadband low-loss passband. First, the middle-layer square patch is replaced by a cross-shaped metal structure with a central slot to enhance the resonance diversity. Second, the element is isolated by a surrounding substrate frame, forming a triple-layer cross-slot configuration with the improved passband characteristics.

The detailed configuration of the triple-layer element is shown in Figure 1. The element consists of three layers. The top and bottom layers (Layer A) are identical 0.508 mm thick square copper plates, each supported by a substrate frame. The middle layer (Layer B) consists of a cross-shaped metal patch and a central slot, also supported by a substrate. These three layers are all supported by the 0.508 mm thick Rogers 5880 substrates with relative permittivity 2.2, loss tangent 0.0009, and dimension 18 mm × 18 mm. The element is symmetrical along both *x* and *y* axes and supports the dual-linear polarization. Here, the element is illuminated by the *x*-direction linear polarized wave.

Each metal layer includes a central cross-shaped slot. A branch slot is added to the end of each arm to expand the transmission phase range. As shown in Figure 1, the element design parameters are: *P* = 18 mm, *w* = 1 mm, *l*_2_ = 1.5 mm. Here, *P* is the element period, *w* is the slot width, and *l*_2_ is the branch length. Parameter *l*_1_ is the length of the cross-slot, which varies from 10 mm to 16.5 mm. Parameter *l*_0_ is the side length of the square metal layer, and *l*_0_ is controlled by *l*_1_ functionally, setting *l*_0_ = *l*_1_ + 0.5 (mm). In Layer B, four square regions are removed from the corners and filled with the dielectric substrate, forming a cross-shaped metal part that encloses the cross-shaped gaps. Parameter *c* is the square side length of the removed square sections in Layer B, and *c* =0.5*l*_1_ − 1 (mm). *l*_1_ ranges from 10 mm to 16.5 mm. The design scales proportionally with parameter *l*_1_ (approximately).

**Figure 2:**
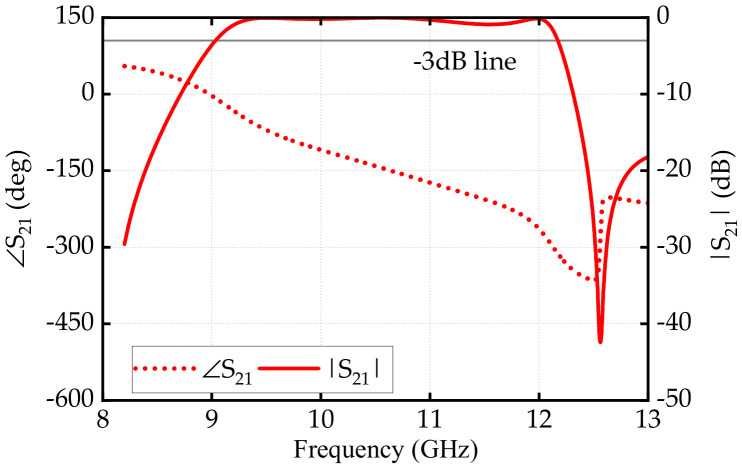
Simulated transmission coefficients of the proposed element without four metal posts (*l*_1_ = 15 mm).

When the parameter *l*_1_ is fixed as 15 mm, the simulated transmission coefficients of the proposed element without four metal posts are shown in Figure 2. Obviously, the proposed element has a wide 3 dB passband of about 3.5 GHz. Since the structure scales approximately with *l*_1_, the broadband operation is preserved across varying element sizes. These results indicate that the modified middle-layer topology together with the isolated configuration is mainly responsible for establishing the broadband low-loss passband of the proposed element. However, according to Ref. [20], the available transmission phase range of this slot-based structure is still insufficient to achieve full 360° phase control.

Therefore, four metallic posts are introduced between the top and bottom layers to further extend the transmission phase range. As a result, a substrate-supported subwavelength transmitarray element with the multi-resonant characteristics is obtained.

**Figure 3:**
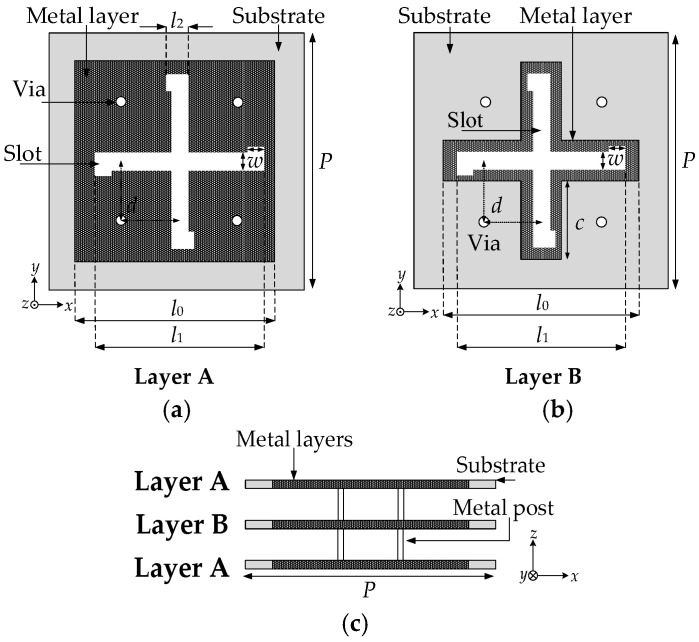
Configuration of the triple-layer substrate-based metal transmitarray element. (**a**) Top view of Layer A; (**b**) top view of Layer B; and (**c**) side view (the element with vias and metal posts).

The detailed configuration of the proposed element is shown in Figure 3. Unlike the element proposed in Figure 1, this element consists of four identical 5.5 mm metal posts that are used to connect the top and bottom layers through vias, penetrating the middle substrate as marked in Figure 3c. The element is symmetrical along both *x* and *y* axes and supports dual-linear polarization. Here, the element is illuminated by the *x*-direction linear polarized wave.

As shown in Figure 3, the element design parameters *P*, *w*, *l*_0_, *l*_1_, *l*_2_ and *c* are the same as those in the previous design shown in Figure 1. Parameter *d* represents the distance between the vias and the element center in the *x* and *y* directions, and *d* = *l*_1_/3 is controlled by *l*_1_ functionally. *l*_1_ ranges from 10 mm to 16.5 mm. The design scales proportionally with parameter *l*_1_.

The proposed element operates based on a multi-resonance mechanism involving two types of resonances, namely slot resonance and current resonance. The slot resonance is jointly contributed by the slots in the stacked FSS layers and the isolated configuration, which introduces additional slot-related capacitive effects between adjacent elements. In contrast, the current resonance is mainly supported by the metallic posts together with the outer conducting layers. The coupling between these resonant responses generates multiple transmission poles within the operating band. As a result, the passband is significantly broadened while maintaining relatively stable transmission amplitude and phase characteristics, which is highly desirable for wideband transmitarray applications.

**Figure 4:**
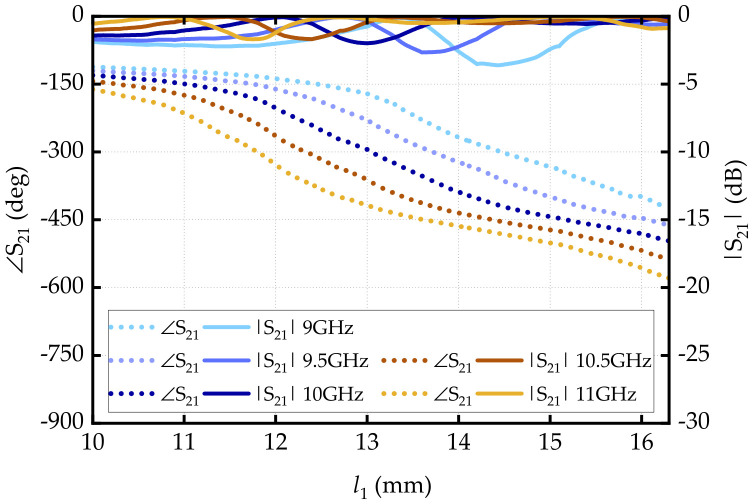
Simulated transmission coefficients of the proposed element at different frequencies.

Next, to achieve a full 360° in the 3 dB passband at the designed center frequency of 10 GHz, four metal posts with the current resonance are introduced. These metal posts pass through the middle layer without interfering with its structure. As shown in Figure 4, at 10 GHz, the transmitting amplitude losses remain below 1 dB across most phase values. Also, the transmitting phase range reaches 360° at 10 GHz. The metal posts with the current resonance increase the element phase range and reduce the transmitting losses. In addition, in the 9–11 GHz frequency band, good phase ranges and low transmitting losses are indicated by the simulated transmission coefficients, since all the curves in Figure 4 maintain their shapes.

To realize the full-phase compensation over a wide frequency band, a phase-partitioning strategy is employed in the array design. The elements are divided into different regions according to their phase response characteristics. Elements with relatively stable transmission amplitude and large phase coverage, e.g., those with *l*_1_ ranging from 14 mm to 16 mm, are allocated in the central region of the array, where the illumination level is higher, while those providing complementary phase responses are placed in the peripheral region. This spatial arrangement ensures continuous 360° phase coverage is achieved while mitigating the performance degradation across the operating band.

**Figure 5:**
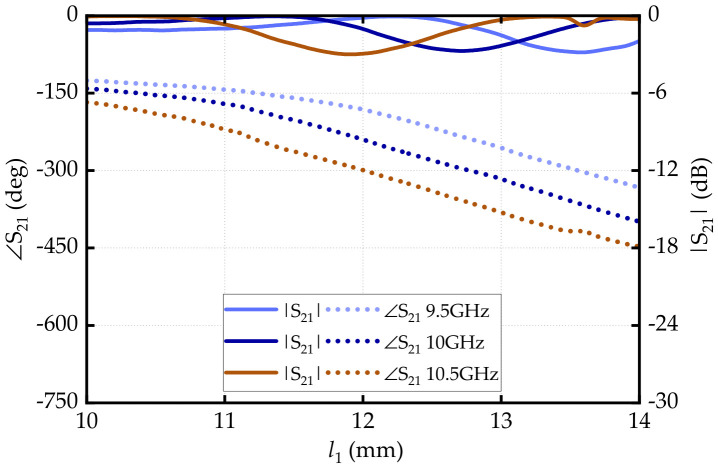
Simulated transmission coefficients of the proposed element when illuminated by a 20° incident plane wave at different frequencies.

Figure 5 illustrates the transmission performance under a 20° incident wave. Such elements are typically placed at the array edges, where the illumination is weaker. Although their contribution is minor, precise phase alignment enhances overall array performance. Thus, edge elements are assigned by a 20° incident wave illuminating situation.

Based on the simulation results, the proposed element is expected to provide precise phase compensation and low transmission losses in the transmitarray applications. Compared with conventional FSS-based triple-layer elements, the use of metal posts significantly broadens the available phase range while maintaining low transmitting losses. Furthermore, the element shows strong potential for high radiation efficiency and flat-gain performance near the center frequency 10 GHz. These characteristics make the element a promising candidate for the design of high-efficiency transmitarray antennas.

Therefore, the proposed design is not introduced as a single combined modification. Instead, the modified middle-layer topology and isolated configuration mainly establish the broadband passband, the metallic posts mainly extend the transmission phase range, and finally, the phase-partition strategy enables the flat-gain performance at the array level.

## 3. Simulation and Measurement of the Transmitarray Antenna

The array synthesis procedure in this work follows the standard phase-compensation framework reported in [20]. For completeness, its implementation in the present design is briefly summarized as follows.

**Figure 6:**
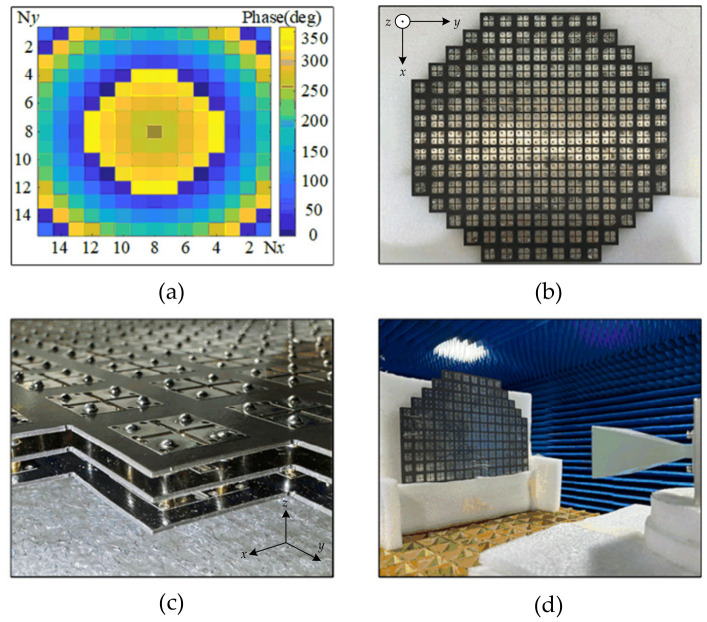
The X-band dual-polarized transmitarray and its far-field measurement setup. (**a**) Phase distribution, (**b**) photograph of the fabricated prototype, (**c**) element structure details, and (**d**) measurement setup in a microwave chamber.

First, an 8.2–12.4 GHz pyramidal horn antenna is used as the feed source, providing an illumination level of −10 dB at the array edges. Based on the −10 dB beamwidth of the horn, a focal-to-diameter (F/D) ratio of 1.2 is selected. With the array size set to 270 mm × 270 mm, the spatial phase compensation is applied to all the elements, as illustrated in the phase distribution shown in Figure 6a.

Then, from an engineering perspective, the simplified fabrication strategy described in [19] is adopted to balance performance and manufacturability. By integrating the element structure with a supporting substrate frame and employing the PCB-based implementation, the mechanical stability of the isolated elements is ensured while significantly reducing the fabrication complexity and cost. Meanwhile, the electrical performance is well preserved, demonstrating the feasibility of the proposed design for practical applications. In each layer, a 0.508 mm thick Rogers 5880 substrate with the double-sided metal etching is used to integrate the metal element with the substrate frame. Additional vias and surface copper layers are employed to ensure electrical continuity. Simulation results confirm that this PCB-based implementation retains a comparable transmission performance.

Following this design strategy, a 185-element octagonal transmitarray with dimensions of 270 mm × 270 mm × 6 mm is fabricated, and photographs of the fabricated prototype and the element structure details are shown in Figure 6b,c. To further enhance bandwidth and efficiency, the initial placement of the center element with *l*_1_ = 15.5 mm ensures the minimal amplitude losses (<1 dB) and linear phase responses within the 9–11 GHz band. Once the central element is fixed, peripheral elements are assigned accordingly based on the desired phase distribution shown in Figure 6a. Then, an X-band dual-polarized transmitarray at the center frequency 10.2 GHz is measured, and its far-field measurement setup in a microwave chamber is shown in Figure 6d.

**Figure 7:**
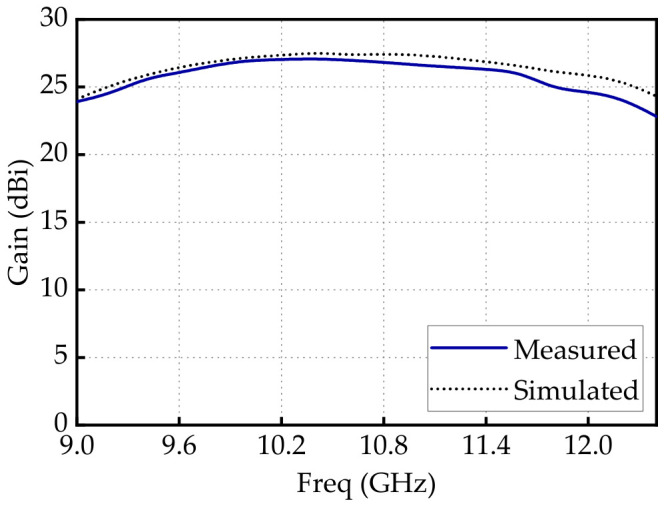
Simulated and measured gain results of the proposed transmitarray.

Figure 7 compares the simulated and measured gain results, which show good overall agreement. Minor discrepancies are primarily attributed to the fabrication tolerances and feed alignment errors. Specifically, the simulated gain reaches 27.56 dBi at 10.4 GHz, while the measured gain is 27.1 dBi at 10.2 GHz. The 1 dB gain bandwidths are 18.9% (9.6–11.6 GHz) in simulation and 19.2% (9.4–11.4 GHz) in measurement, while the 0.5 dB gain bandwidths are 13.2% and 12.7%, respectively.

The fabrication tolerances and feed position errors may result in a greater impact on the array center frequency, which deviates from 10.4 GHz to 10.2 GHz, with the increased transmission losses. Small deviations in the layer registration, geometric dimensions, and feed positioning may jointly influence the measured response. Among them, the structural tolerances mainly affect the resonance location and transmission phase, whereas the feed-position uncertainty mainly perturbs the aperture phase-compensation condition. Their combined effect is believed to be responsible for the observed shift in the gain and the slight reduction in the measured gain. Therefore, the observed frequency shift is mainly caused by structural deviations and feed positioning inaccuracies, which tend to have a greater impact near the resonant center frequency. In contrast, their influences are less pronounced at the band edges, leading to a broader measured 1 dB gain bandwidth despite a slight drop in the peak gain. Benefiting from the precise phase control and the low-loss element design, the proposed transmitarray achieves a measured radiation efficiency of 58.9% at 10.2 GHz.

**Figure 8:**
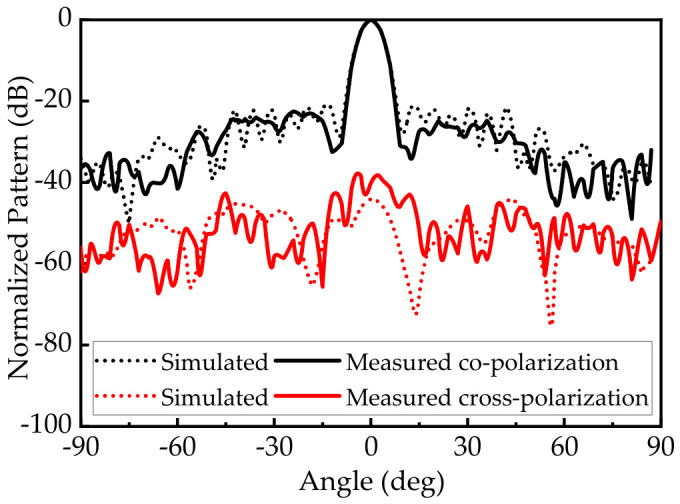
Simulated and measured *xoz*-plane radiation patterns at 10.2 GHz.

Simulated and measured *xoz*-plane radiation patterns of the proposed transmitarray at 10.2 GHz are presented in Figure 8, demonstrating strong consistency. The sidelobe levels are suppressed below −20 dB. With the polarization isolation exceeding 35 dB and excellent pattern symmetry, the design confirms robust dual-polarization capability.

**Figure 9:**
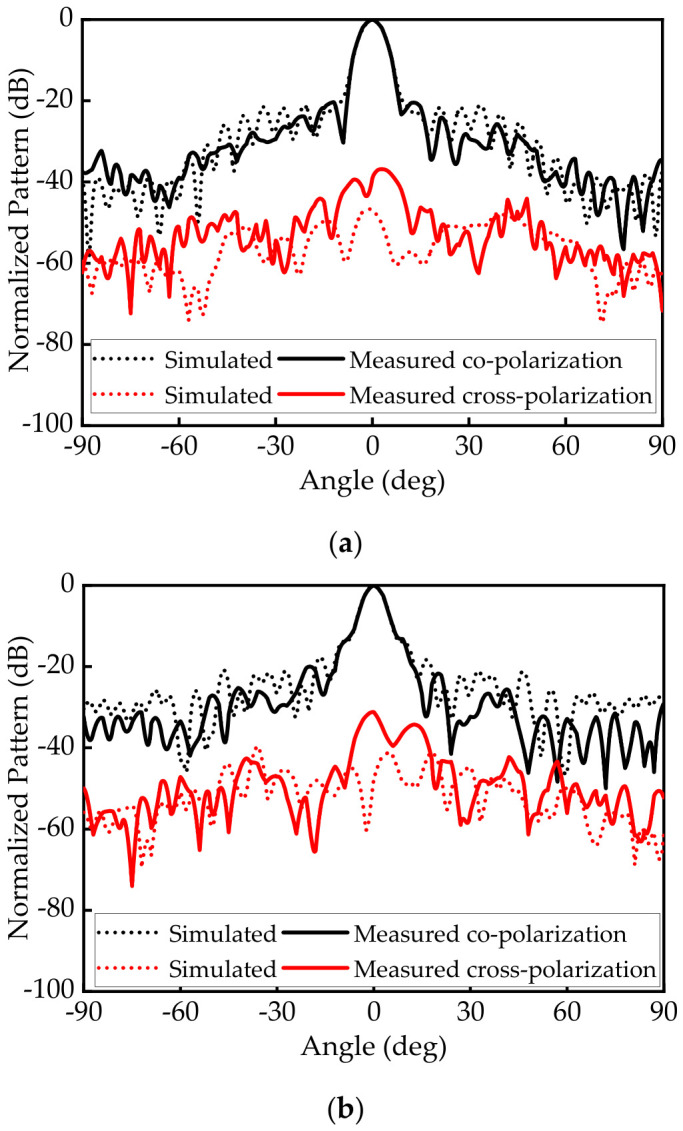
Simulated and measured *xoz*-plane radiation patterns at two 1 dB side frequencies: (**a**) 9.4 GHz and (**b**) 11.4 GHz.

In addition, Figure 9 presents the measured radiation patterns at the 1 dB band edges (9.4 GHz and 11.4 GHz). The patterns maintain desirable shape and high directivity, with a minimum radiation efficiency of 37% achieved at 11.4 GHz, confirming effective broadband operation. Here, the radiation efficiency is obtained from the measured gain and the physical aperture area of the transmitarray using the conventional radiation efficiency expression

$$\eta =\frac{G\lambda^{2}}{4\pi A}$$

where *G* is the gain, *λ* is the free-space wavelength, and *A* is the physical aperture area.

Finally, Table 1 compares the proposed design with some other dual-polarized wideband transmitarrays and single linear-polarized flat gain transmitarrays reported in the literature. Obviously, the proposed design offers superior radiation efficiency and a wider flat-gain bandwidth while supporting the dual polarization characteristics.

## 4. Conclusions

In this work, a wideband triple-layer cross-slot element with multiple resonances is developed, incorporating a cross-shaped middle layer and four vertically connected metal posts to extend the transmission phase range and enhance the radiation efficiency. Based on the proposed subwavelength element, an octagonal dual-polarized transmitarray with 185 elements is designed, fabricated, and measured. The measured results confirm that the transmitarray achieves high radiation efficiency (58.9%), a flat-gain response (0.5 dB bandwidth of 12.7%), and a wide 1 dB gain bandwidth (19.2%) centered at 10.2 GHz.

These results demonstrate the effectiveness of the proposed element design and its suitability for the high-gain, dual-polarized applications in the X-band wireless communication and radar systems. Compared with previous designs, the proposed approach provides a practical route to simultaneously achieve dual polarization, high efficiency, and flat-gain performance. The results demonstrate that the integration of multi-resonant element design and phase-partition-based array arrangement is effective for controlling the gain variation across frequency.

## Figures and Tables

**Table 1:** Comparison of the proposed design with some existing DP wideband and single LP flat-gain transmitarrays.

Ref.	Gain Bandwidth	Eff.	Gain (dBi)/ Freq. (GHz)	Size (*λ*^2^)/ Profile (*λ*)	Pol.
[5]	37.0% (1 dB)	25.6%	22.5/10	6.5 × 8.5/ 0.347	LP
[6]	16.9% (1 dB)	61.0%	22.1/9.4	5 × 5/ 0.164	LP
[7]	41.3% (0.5 dB)	32.5%	25.0/11	8.8 × 8.8/ 0.22	LP
[8]	24.27% (1 dB)	62%	29.2/14.3	5 × 5/ 0.462	LP
[14]	5.7% (1 dB)	41%	23.0/9.7	6.2 × 6.2/ 0.033	DP
[15]	9% (1 dB)	30%	28.9/11.3	8.1 × 8.1*π*/ 0.509	DP
[18]	20.4% (1 dB)	52.5%	27.1/12	8.8 × 8.8/ 0.12	DP
[19]	10.3% (0.5 dB)/	56.1%	26.9/10.2	9.2 × 9.2/ 0.204	DP
This work	**12.7%** **(0.5 dB)/** **19.2%** **(1 dB)**	**58.9%**	**27.1/10.2**	**9.2** **× 9.2/** **0.204**	**DP**

Here, λ is the wavelength corresponding to the center frequency; Eff. stands for radiation efficiency; Freq. stands for frequency; Pol. stands for polarization; LP stands for linear polarization; DP stands for dual-polarization. Profile stands for the array profile height without feed.

## Data Availability

All data generated or analyzed during this study are included in this manuscript. There are no additional data or datasets beyond what is presented in the manuscript.
